# The views of the general public on prioritising vaccination programmes against childhood diseases: A qualitative study

**DOI:** 10.1371/journal.pone.0197374

**Published:** 2018-06-13

**Authors:** Gemma Lasseter, Hareth Al-Janabi, Caroline L. Trotter, Fran E. Carroll, Hannah Christensen

**Affiliations:** 1 Population Health Sciences, Bristol Medical School, University of Bristol, Bristol, United Kingdom; 2 Institute of Applied Health Research, University of Birmingham, Birmingham, United Kingdom; 3 Department of Veterinary Medicine, University of Cambridge, Cambridge, United Kingdom; 4 Royal College of Obstetricians and Gynaecologists, London, United Kingdom; 5 Population Health Sciences, Bristol Medical School, University of Bristol, Bristol, United Kingdom; Public Health England, UNITED KINGDOM

## Abstract

**Background:**

Decisions regarding which vaccines are funded in the United Kingdom (UK) are increasingly informed by cost-effectiveness analyses. Such analyses use Quality Adjusted Life Years (QALYs) as a measure of effectiveness and assume that QALYs are equal regardless of where and in whom they occur in the population. However, there is increasing debate about whether this QALY approach is appropriate and whether societal preferences for childhood vaccinations should be used to help inform childhood immunisation policy.

**Objective:**

To gauge the general public’s preferences for prioritising certain characteristics of childhood vaccination, to help inform future policy making decisions in the UK.

**Design:**

Qualitative design using individual face-to-face interviews, with data analysed using an inductive thematic framework approach.

**Setting:**

Two counties in England, UK.

**Population:**

Adult members of the general public were recruited using the Bristol and South Gloucestershire open electoral registers, using gender and deprivation quotas for each area.

**Participants:**

21 members of the public participated in qualitative interviews.

**Results:**

The qualitative research identified three major themes and several key attributes that influences participant’s opinions about priority setting for childhood vaccinations: (1) population segment (i.e. age group, carer impact and social group), (2) vaccine preventable diseases preferences (i.e. disease severity, disease incidence and declining infection) and (3) risks and benefits associated with childhood vaccinations (i.e. vaccine associated side-effects, herd protection and peace of mind).

**Conclusion:**

Evidence from this qualitative study suggests that some members of the UK general public have more nuanced views than the health-maximisation approach when considering how childhood vaccines should be prioritised. This is not necessarily captured by the current economic approaches for assessing the benefits from childhood vaccinations in the UK, but is an important area for future research to ensure appropriate decision making.

## Introduction

Cost-effectiveness is a crucial consideration for vaccination programmes in the United Kingdom (UK), as shown by the recent debate and policy decision around the introduction of Bexsero^®^ to protect against meningococcal group B disease[1]. Cost-effectiveness analyses consider the net cost of the intervention against the net health gains that result. The preferred measure of health gain used by National Institute for Health and Care Excellence (NICE) and the Joint Committee on Vaccination and Immunisation (JCVI) is the QALY or quality adjusted life year[2]. The QALY combines both the length of life and the quality of life a person experiences during that time; one QALY is equal to one year of life in perfect health.

Current economic analyses are conducted on the premise that all QALYs are equal, for example a QALY gained by a child has the same value as a QALY gained by an adult, or for any other person in society regardless of demographic factors, background or circumstance[3]. This methodology assumes that a single person gaining a large improvement in their health is the same as several people gaining a small improvement in their health[3]. However, vaccines as a health intervention, particularly in children, are distinctive as they are often given at a very young age to confer protection into adulthood, thus in this group there may be particular reasons to reject the conventional wisdom that a “QALY is a QALY is a QALY”. Indeed, the QALY approach for assessing the benefit from childhood vaccination has been challenged recently because it does not take into account special consideration of factors and thus could fail to take into account social preferences[4–7]. For example, people may want to prevent rare severe diseases more than common mild ones, may fear certain diseases, and may place particular value on the life-saving preventative nature of vaccination. There have been attempts to address these issues by defining weights for use in health care[8–12]. These studies, although not solely focused on the benefits associated with childhood vaccines, have shown that society has stronger preferences for health gains in younger people, those who are critically ill and the socioeconomically disadvantaged[8–12]. Other DCE studies and surveys have considered societal preferences related to health interventions[13–16], nevertheless there remains a paucity of evidence within the UK about whether current policy decisions represent public opinion[17], indeed the question of whether the current QALY approach truly reflects UK societal preferences has not been formally addressed. This study aimed to identify public preferences for the health gains associated with vaccination and assess whether the current assumption of equal QALY values is valid when considering childhood vaccination programmes in the UK.

## Methods

### Study design

An explorative qualitative design using individual, face-to-face interviews with members of the public. COREQ guidelines have been used in reporting the study conduct and findings.[18]

### Setting and participants

**Fig 1:**
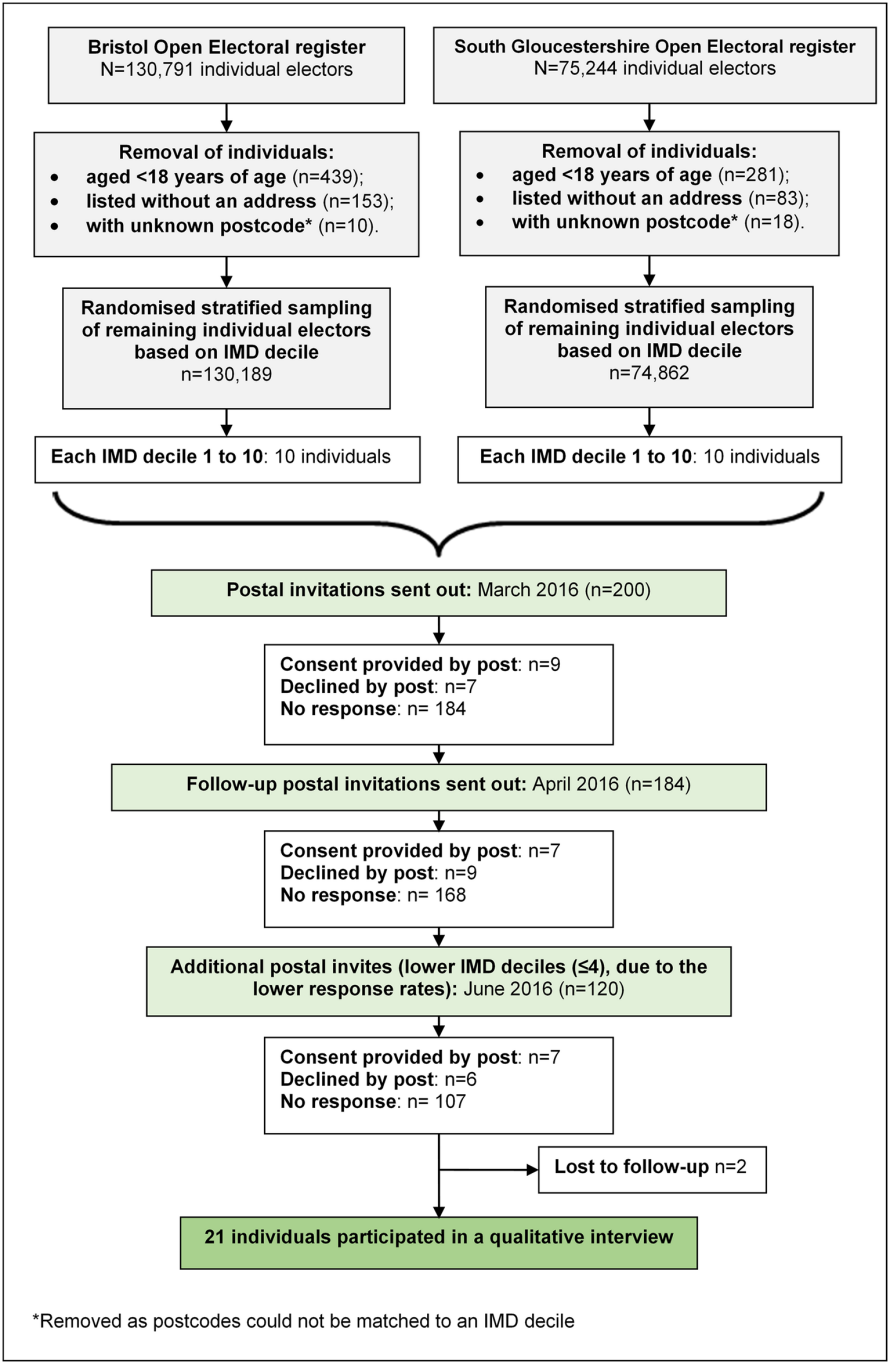
Recruitment flow diagram for qualitative interviews.

We recruited members of the public from two counties in South West England. These counties were chosen after reviewing the annual (2014–2015) vaccination coverage statistics for children aged up to five years in England for Bath and North-East Somerset, Bristol, Gloucestershire, North Somerset and South Gloucestershire[19]. Vaccine uptake in children up to the age of 5 was found to vary the most between Bristol (generally lower) and South Gloucestershire (generally higher) during this period, therefore these areas were chosen to ensure a population with some diversity in vaccination uptake would be included in the study. Electronic copies of the open electoral registers for Bristol City Council and South Gloucestershire City Councils were obtained and used to identify individuals registered in each district. After data cleaning, 130,791 and 74,862 individuals were listed on the Bristol and South Gloucestershire City Council electoral register respectively (Fig 1). Postal qualitative interview invitations were sent to a stratified random sample (based on Indices of Multiple Deprivation decile) of adult individuals (≥18 years) recorded on each register.

Potential participants received an English language information sheet describing the purpose and nature of the research and a sheet in ten languages (Arabic, Chinese, Gujarati, Hindi, Kurdish, Polish, Portuguese, Punjabi, Somali, Urdu) inviting them to take part in the study and stating that full information in one of the ten languages could be provided. Interested individuals were asked to return their written informed consent to the University of Bristol study team if they were willing to participate in the research.

### Data collection

Interviews were conducted by the corresponding author G.L. (female/PhD/senior researcher). G.L. had previously received qualitative interview training and was experienced in conducting interviews with members of the public. Each participant was interviewed on one occasion and knew that the research was contributing towards childhood vaccination research. Interviews were arranged at a time convenient to the participant and conducted in a venue of their choosing (e.g. their home or local coffee shop). No individuals other than G.L. and the participant were present during the interviews. It was emphasised throughout each interview that participants could stop the interview or withdraw from the study at any point. A topic guide was used to ensure participants’ opinions were investigated fully during the interview (see below). Each interview lasted one hour on average and was audio recorded, with retrospective field notes created as required. Each interview was transcribed verbatim shortly afterwards; all data were subsequently anonymised. Each participant received a £10 shopping voucher for their participation.

### Interview topic guide

A semi-structured interview topic guide was used, this ensured that fixed themes were discussed during each interview, but also allowed participants to discuss themes that they personally considered to be pertinent. Each interview started with some ‘background mapping’ questions[20] about the participant and their general views about childhood vaccinations, before moving onto questions about specific health benefits that participants associated with vaccination (S1 File). The main topic for discussion was the different types of attributes that policy makers should consider when funding childhood vaccination programmes, including factors related to disease, the vaccine and who receives the benefit.

Each interview began with two vignettes, which were developed with input from all members of the study team (discussed below). These ‘snapshot’ scenarios were used so that two vaccine preventable disease stories unfolded through a series of stages.[21] This ‘continuous narrative’ approach [22] was chosen to encourage participants to comment at each stage of the story and to discuss their own beliefs and opinions about a topic that they potentially had very little knowledge or experience about. Follow-up questions, probes and qualitative methods were used to encourage participants to express their views, these methods included, but were not limited to: flash-cards, think-aloud techniques and open discussion. In later interviews, as key themes were identified, the topic guide was updated (S2 File) so that the concluding section of each interview was used to confirm the importance of key themes and to explore if they should be considered for inclusion in a subsequent DCE questionnaire survey; this approach allowed suggestions from preceding interviews to be tested with latter participants.

### Analysis

An inductive thematic framework approach[23] was used to analyse batches of between three and six interview transcripts. Analysis began with thoroughly reading through each transcript whilst listening to the accompanying audio file; this ensured that all verbal emphases were captured during the analysis process. After this initial reading of transcripts, a general coding structure was developed from the data and applied to large sections of text, then paragraphs and finally to particular sentences. New codes were developed and applied as necessary, with repeated review of the transcripts to ensure consistency in the application of the coding scheme. All transcripts were reviewed and compared multiple times until no new codes were identified[24]. During the coding process the synthesised data were used to generate descriptive accounts in order to identify key attributes and to map the range and diversity of participants’ opinions[20]. During this analysis process, the preliminary results were discussed by the whole study team, which assisted in the development of themes and sub-themes. Analysis of the interview transcripts continued until saturation was achieved, whereby no new themes were identified.

Data analysis was primarily conducted by G.L. with support from an experienced research team (H.A.-J., C.L.T., F.E.C. and H.C.) with a range of expertise; (H.A.-J.) health economist with qualitative research experience and DCE development experience, (F.C.) health psychology with qualitative research and DCE development experience, and (C.L.T and H.C.) epidemiologists. The breadth and range of experience within the research team ensured that different opinions were discussed during the data interpretation process and the resulting coding framework was used after being approved by all team members.

Four rounds of interviews were conducted in total, this allowed the study team to meet and discuss the results before moving on to the next round of interviews. The terminology used by respondents during the first round of interviews was used to help explain the information presented to participants in subsequent interviews. The final round of interviews was used to check for saturation and confirmation of the overall findings.

The whole interview and analysis process was iterative, with the topic guide and key attributes updated using suggestions identified during each round of interviews. Analysis of the qualitative interview data was managed using NVivo version 10[25], which facilitated organisation of interview transcripts and linkage with participants’ demographic data.

Ethical approval for this study was obtained from the University of Bristol, Faculty of Health Sciences Research Ethics Committee in Bristol, England (application number 29821).

## Results

Interviews took place between March and December 2016. Twenty-three individuals expressed an interest to participate, with a total of 21 (17 women, 4 men) finally included in this study. Two potential participants were lost to follow-up after researchers were unable to re-contact them to arrange an interview after multiple attempts.

**Fig 2:**
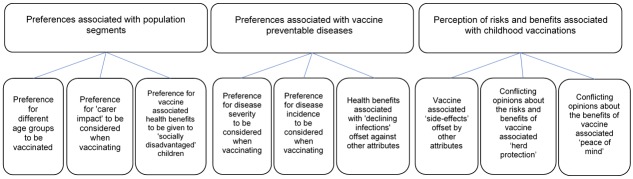
Themes and categories that emerged during analysis. Qualitative themes are presented in the top row and categories in the bottom row.

Characteristics of participants covered a broad set of ages and parental categories (Table 1; additional characteristics are provided in the S3 File). Interviews lasted between 33 and 70 minutes with a mean duration of 58 minutes. The thematic qualitative analysis process resulted in three main themes: social preferences associated with childhood vaccination priority setting, preferences associated with vaccine preventable diseases, and perception of risks and benefits associated with childhood vaccinations. Each of these themes includes three categories (Fig 2).

**Table 1:** Descriptive characteristics of participants (n = 21).

Characteristics	Number
Location	
South Gloucestershire	9
Bristol	12
Age range of participants (years)	
35–44	6
45–54	4
55–64	3
64–74	6
75+	2
Sex	
Female	17
Male	4
Ethnicity	
White British	20
White European	1
Parental status	
Not a parent/guardian	6
Parent/guardian	13
Parent/guardian and foster carer	2
Highest qualification	
A-level or equivalent	2
HNC or equivalent	4
Degree or equivalent	8
Doctorate or equivalent	3
No formal qualifications	4

### Preferences associated with population segments

#### Preference for different age groups to be vaccinated

Many of the interview participants were in favour of funding vaccination programmes that targeted specific age groups. This was because age was considered as a proxy for vulnerability to disease, with the very young (≤ 1 years) and elderly in society perceived as being more susceptible.

I think 1 year old or less is a vulnerable age group from health wise and you know within considering possible death, causes of death. Once they get a bit older, say two year olds, they’re a bit more robust…

(Male, parent/guardian, interview #16)

The vulnerability of these two age group extremes seemed to be accentuated within the youngest age group, with the belief that diseases were more readily contracted and developed more quickly in younger children.

[S]mall children become unwell very quickly, very quickly indeed. Erm I’m not saying adults don’t but certainly children, you can see them dramatically go downhill within hours.

(Female, not a parent/guardian, interview #7)

Further investigation around the age theme showed that some participants made decisions about rationing vaccinations based on the amount of life remaining, with some making judgements about what constituted a ‘fair-innings’. In these instances, some participants believed that younger members of society would enjoy the protection from a vaccination for longer than their older counterparts and consequently vaccination programmes should be funded to maximise these health benefits.

I’d rather help the young ones to have a longer life that was healthy…I think that the young people that have got a long time left, then there’s more value to it for them. So I’m not saying that you’re not valuable when you’re an older person but… it’s a longer time that they’ve got to live, hopefully, healthily.

(Female, parent/guardian, interview #2)

#### Preference for ‘carer impact’ to be considered when vaccinating

Some participants mentioned the effect that vaccine preventable disease had on the quality of life for those responsible for caring for a child suffering with vaccine preventable disease. This ‘carer impact’ was thought to be felt most acutely by close family and friends and would affect their emotional health and wellbeing whilst caring for a child during and after their illness.

[J]ust considering the impact on families or, children with diseases that they could’ve been immunised against…it’s a way of making policymakers think about the effects, because they tend to think in financial terms, not necessarily in effects on the family, on parents, on siblings and psychological effects…depression, anxiety, self-harming, any, any of those kind of things that can be triggered by extreme circumstances.

(Male, parent/guardian, interview #16)

Taking ‘carer impact’ into consideration when making funding or policy decisions about childhood vaccinations was considered essential, especially since this burden could have both financial and psychological effects throughout the wider family unit. Nevertheless, participants generally thought that the short-term care associated with looking after an ill child could largely be coped with by family units, whilst the burden of long-term care had emotional and financial impacts that should be avoided if at all possible.

I think you know as a, whatever, family unit you’ve got um, getting through that short-term care burden is a lot easier than that long-term care burden, everything, emotionally, care itself, financially. Um, so yeah definitely I think, and again, that must be a consideration for the government in terms of how much money, um it costs to support a child and a family with long term care needs and verses the cost of the vaccination.

(Female, parent/guardian, interview #18)

#### Preference for vaccine associated health benefits to be given to ‘socially disadvantaged’ children

Some participants were in favour of using socio-economic status to help inform funding decisions about publicly funded childhood vaccination programmes. These opinions were often based on the belief that there was a social dichotomy in the UK, whereby families (or more specifically children) could be classified into either a socially advantaged or socially disadvantaged group, with children in the socially disadvantaged group having lower underlying health status and therefore their preference would be to target any health benefits to these individuals.

I think probably they [socially disadvantaged children] haven’t got the food going into them that the well-off people have, so I think they need it [vaccinations] more, they wouldn’t be able to cope with the illness, I don’t think they would survive so easily.

(Female, not a parent/guardian, interview #13)

Overall, participants found it difficult to decide how health benefits might be fairly distributed in society and struggled with the ethical dimensions associated with this issue. When discussed further it became clear that some participant’s attitudes about social advantage/disadvantage and rationing health benefits were often intertwined with perceptions of wealth; whereby those children in the socially disadvantaged group would have fewer opportunities for improving their underlying health.

I wouldn’t be able to differentiate between either [socially advantaged or disadvantaged children] because a human being is a human being. Err I, I don’t—I wouldn’t be able to differentiate between them. If, if I had to I would say, erm possibly the poorer people in that they don’t have the ability to possibly change their lifestyle. So presuming that lifestyle had something to do with the illness, a rich person might be able to help themselves. That’s my only reasoning there.

(Female, parent/guardian, interview #7)

Conversely, a few participants strongly believed that health benefits should be offered to all children in the UK, irrespective of their socio-economic status.

I still think it should be absolutely across the board, whoever you are, if you’re aged two and you get a vaccine, it doesn’t matter what socioeconomic status or where you live in the country or if you’re in a high rise flat or your mum shops at Iceland or whatever it is, you as that child should be included in that vaccination programme come hell or high water.

(Female, not a parent/guardian, interview #21)

### Preferences associated with vaccine preventable diseases

#### Preferences for disease severity to be considered when vaccinating

A range of disease severity was identified, with the general consensus in favour of prioritising vaccinations against severe diseases that might cause long-term health implications, whilst milder self-limiting diseases were thought to be less important to vaccinate against.

If it’s something that you can get over, without any long-term consequences, then I’m not sure there’s a real need to be vaccinated against it. But if it could result in death or long-term health consequences then I think it’s a different case.

(Female, parent/guardian and foster carer, interview #6)

Well, if it’s going to kill you without a shadow of a doubt there should be a vaccine for it. If it’s going to disable you there should be a vaccine for it. If it’s gonna give you a sneeze, you could consider whether or not to vaccine against it. So it depends on the effect on mortality and the effect on potential creation of life-limiting conditions and potential disability.

(Female, not a parent/guardian, interview #21)

Focusing discussions around the impact of vaccine preventable diseases on children meant that participants freely discussed the issue of long-term consequences as a key concern.

Because, you know, if, if you get a cold, or a fever, or whatever, you can just fight back from that. But the long-term health implications, you know, could affect their futures, could affect how they get jobs in the future, could affect their life and then you’ve got the affects [on] their families.

(Female, not a parent/guardian, interview #13)

#### Preferences for disease incidence to be considered when vaccinating

Discussions around the severity of disease often led to participants also considering the significance of disease incidence, as these two sub-themes were frequently interlinked. As with severity, variations in disease incidence were discussed by participants, ranging from rare to common. Participants expressed a preference for prioritising childhood vaccinations against common diseases, as this would represent better value for money.

[I]f it was only going affecting a few people, then…and it was, it was only mild and it was only a week, then you know, your money might be better off spent on something which is a bit—affects more people and has wider consequences.

(Female, parent/guardian, interview #18)

#### Health benefits associated with ‘declining infections’ offset against other attributes

Participants were asked to think about whether childhood vaccinations should continue to be funded for diseases that were declining. However, there were numerous comments concerning the complexity of this issue, as it was felt that the reason for the decline needed to be identified before a definitive decision could be made.

If it’s declining as a result of the vaccination policy then that’s absolutely not the point at which to stop the vaccination policy…if it’s declined because it was the result of eating, I don’t know, clam shells, and nobody eats clam shells anymore, then fine.

(Female, parent/guardian, interview #17)

Despite the complexity of the topic, most participants commented that declining diseases should continue to be vaccinated because of the need to ensure that the disease does not re-emerge in the future.

I think it’s really important that we continue funding vaccinations because, until you’ve proved that the disease doesn’t exist in the world anymore, you’re effectively stopping funding the reasons for your success.

(Male, parent/guardian, interview #16)

### Perception of risks and benefits associated with childhood vaccinations

#### Vaccine associated ‘side-effects’ offset by other attributes

The potential side-effects associated with childhood vaccinations were a key issue for some participants, as there were some concerns that the consequences of having a vaccination could potentially be worse than contracting a disease.

Oh yes, side-effects are very important. Sometimes they’re worse than the disease and they would have to be considered.

(Female, parent/guardian, interview #12)

However, numerous participants often discussed this concern in the context of the disease that could be prevented and considered the potential for vaccine associated side-effects against other attributes. These individuals believed that disease severity and incidence played a bigger role in the funding decisions related to any national childhood vaccination programme.

[I]t’s very subjective, so it would depend on the consequences of the diseases that you’re being vaccinated against, weighed up against the side effects and the percentage chance of being infected by the disease and the percentage risk of suffering from the side effects. So it would be sort of fairly complex decision tree based on those factors, I think.

(Male, parent/guardian, interview #16)

#### Conflicting opinions about the risks and benefits of vaccine associated ‘herd protection’

The concept of herd protection was explained to all participants during the interview process and the ensuing discussions showed that most individuals considered this an important attribute in relation to prioritising funding for childhood vaccination programmes.

I would say I would favour the herd vaccination…because, erm, potentially much greater benefits. Similar level of benefit for the individual, but a much greater level of benefit for the collective.

(Male, parent/guardian, interview #16)

Yet some participants were concerned about the risks associated with vaccinations and felt that the health and safety of their own children was more important than seeking a vaccination that might confer herd protection and contribution to the health of the population.

[I]f everybody else has been vaccinated so that the disease isn’t prevalent, mine aren’t gonna get it and I don’t have any risk of any of the possible complications, whether or not they exist. So my core prejudice has always been to avoid any form of medication or interference, but I’ve actually followed social rule and just gone and had it [vaccinations] done

(Female, parent/guardian, interview #17)

The social acceptability of childhood vaccinations that conferred herd protection was an issue for several participants. This belief was aired from an individual perspective, but may also reflect a more collectively shared opinion regarding the benefits of vaccine associated herd protection.

Well I do think that the whole way in which immunisation programmes work to protect whole societies is a really crucial aspect of their benefits, so being able to think beyond your own individual wellbeing is fundamental to living in a good society.

(Female, parent/guardian, interview #4)

#### Conflicting opinions about the benefits of vaccine associated ‘peace of mind’

Some of our participants suggested that the reassurance, or ‘peace of mind’, associated with childhood vaccinations was a potential benefit worthy of consideration. These findings showed that reducing anxiety might have a role to play in increasing an individual’s intention to vaccinate and perhaps such preferences might need to be considered by policy makers when funding childhood vaccination programmes in the future.

I think a lot of people do worry a lot and I think it can make their lives miserable…I think some people do get a certain sense of ‘done that [vaccination], so I should be all right now’. If that makes them go out into the world feeling better…so peace of mind is always a good investment.

(Female, parent/guardian, interview #17)

The peace of mind concept was also associated with a feeling of parental responsibility, whereby failure to fully vaccinate children could be considered socially unacceptable or irresponsible.

Yeah, I think peace of mind as a parent is, is very important. Knowing that your child has got a tick in all the, all the vaccine boxes that it should have. I think that’s, yeah, that’s something that every parent should be looking out for and until you, until you’ve actually made those arrangements and had those vaccinations given, you’ve fallen short in your parental obligations.

(Male, parent/guardian, interview #16)

Conversely, a couple of participants believed that peace of mind should not be taken into consideration when making decisions about childhood vaccinations. These individuals appreciated that emotions might motivate some individuals in society to seek childhood vaccination; nevertheless, it was considered unfitting for policy makers to make funding decisions from a vaccine associated peace of mind perspective.

Well no, because there can be totally irrational fears like the whole MMR irrational thing, um where no, no, no, no terrible to have an immunisation policy based on people’s emotional feelings towards it.

(Female, parent/guardian, interview #4)

I don’t think that should come into it. I think that’s just for erm, individual’s peace of mind and you can’t, you can’t vaccinate for that reason. You can only vaccinate to, to stop the spread of diseases. You can’t put that in the equation, I don’t think.

(Female, parent/guardian, interview #11)

## Discussion

### Principal findings

We found that members of the public have preferences regarding the types of features that should be taken into consideration when making funding decisions about childhood vaccinations in the UK. Three main themes including nine key attributes influenced interview participants’ opinions. These were: (1) preferences associated with population segments, which were linked to age group, carer impact and social group, (2) preferences associated with vaccine preventable diseases, which were attributed to disease severity, disease incidence and declining infection, and (3) perception of risks and benefits associated with childhood vaccinations, which were linked to vaccine associated side-effects, herd protection and peace of mind. Overall, participants demonstrated support for using criteria that both fall within and outside the cost per QALY calculations. These findings indicate that the current economic analysis approach, which assume that all QALYs are equal, may need to be refined when considering childhood vaccinations.

### Strengths and weaknesses of the study

To our knowledge this is the first study to explore societal preferences for childhood vaccination priority setting in the UK. The qualitative interview data were obtained from members of the public from two counties with differing vaccination uptake rates, which improves the generalisability of our study population. A weakness of this study was the sampling methods used; 504 individuals were invited to take part in the study using information recorded on the open electoral registers for Bristol and South Gloucestershire, but only 21 individuals eventually participated in the study. The open register is an extract of the electoral register and as such is an incomplete list of electors and, moreover, does not list adults who are not eligible to vote. Therefore, it is possible that our results do not truly represent the diversity of opinion within the UK. Nevertheless, the broad range of preferences identified during the face-to-face interviews and the fact that data saturation was achieved would indicate that the qualitative results presented provide an in-depth insight into the preferences of those participating. Analysis of our qualitative data indicated that we recruited individuals with a range of opinions on vaccinations; individuals that were well informed about vaccines, individuals who were largely unfamiliar with the topic and individuals with a range of opinions about vaccinations (anti-, pro- and neutral-vaccine opinions). Nevertheless, purposefully recruiting a more diverse range of qualitative interview participants may have provided a wider insight into current public opinion and inclusion of some participants from non-white British ethnic backgrounds would have been preferable.

### Comparisons with existing literature

Our findings, that members of the public in the UK have preferences for attributes related to who should be vaccinated and under what circumstances, are supported by other studies in this area. Age (especially the youngest in society) was thought to be associated with disease vulnerability and thus felt to be a key priority group for vaccinations. A systematic review by Gu et al (2015) also found that the youngest in society were often favoured when contemplating health care priorities[26]. Age is implicitly included in the current QALY calculation as those vaccinated at younger ages have more years of benefit ahead of them; further work is required to estimate whether additional age-weighting is required.

Participants also discussed the ‘carer impact’ of diseases, which they believed should be considered when making childhood vaccination funding decisions. NICE methodology already recommends that ‘[a]ll direct health effects, whether for patients or, when relevant, carers’ should be included in assessments[27] and whilst carer QALYs have been included in some cost-effectiveness analyses, this approach is not done routinely and when implemented does not form the base case. The rationale for excluding carer QALYs is often multifaceted, but primarily results from a lack of data in some fields and the desire to ensure a consistent approach with previous work. Therefore, unanswered questions remain about whether additional weighting is required when considering the severity of the ‘carer impact’, in the same way additional weighting may be required for disease compared to mild illness in persons directly affected.

Some participants had clear notions of preferences associated with particular population segments when prioritising childhood vaccinations based on social group, with socially disadvantaged children given precedence over socially advantaged children. This is in line with previous research studies considering public attitudes towards health inequalities and healthcare priority setting, which found that members of the public tended to favour individuals with low socioeconomic status (SES) compared to those with a high SES score for health care interventions[28–33]. Our findings also showed that participants had preferences associated with disease related attributes, such as severity, incidence, and infection trends over time. These findings add to the findings of Nord et al (2014 and 2015), who identified disease severity as one of the most important attributes for consideration when setting health care priorities[34,35].

The perception of risk and benefits associated with childhood vaccinations also influenced our participants’ opinions. Discussions centred on vaccine associated side-effects, herd protection and peace of mind, although these preferences were often considered alongside other factors (e.g. such as disease severity) as part of the decision-making process. Side-effects were a common concern raised during the interviews, whereby participants were less likely to prioritise vaccinations that might cause harm to children. A similar finding was reported by Sadique et al (2013), whereby the severity of vaccine associated side-effects influenced mothers’ decision making processes about vaccinating their children, although the probability of these side-effects occurring was not a consideration[36]. Side-effects were also considered by our study participants in conjunction with herd protection, as those who believed vaccinations were unsafe were less likely to accept the risk of vaccination in order to potentially protect others from infection. This hesitation was akin to the public opinions reported by other previous UK qualitative studies, which focused on parental decisions about MMR vaccinations[37, 38]. This idea of parental decisions and ‘anticipated regrets’ associated with vaccinations was also discussed by Sadique et al (2013), an idea that seems closely akin to the ‘peace of mind’ attribute identified during our qualitative study[36]. These anticipated regrets were described as a trade-off between the risks associated with vaccine associated side-effects in instances when a vaccination was taken, balanced against the potential for catching a vaccine preventable disease in instances when a vaccination was not taken. This trade-off approach was similar to that taken by participants in our study, who often made decisions by offsetting the benefits associated with ‘peace of mind’ against other preferences, such as disease severity[26, 36, 39, 40].

### Meaning of the study: Possible explanations and implications for clinicians and policy makers

Our findings show that the general public consider a range of population segment, disease and vaccine specific attributes when evaluating the relative importance of different childhood vaccination programmes. These preferences include many attributes that would enter into a cost-per QALY calculation, but may also go beyond the current aspects considered in the standardised approach recommended by NICE for technology assessment economic evaluations. For example, a preference for vaccinating younger rather than older individuals, which is greater than currently accounted for by the standardised approach. Thus it would seem that the public may not consider QALYs gained to be equal across and between people for a number of attributes. Policymakers considering introducing new vaccine programmes, or modifying existing ones, should consider whether, and how best, to include such societal preferences into their decision making.

### Unanswered questions and future research

Our findings suggest that some members of the UK general public have more nuanced views than those allowed by the health-maximisation approach when considering how childhood vaccines should be prioritised. Our participants identified several attributes that they believed policy makers should consider when making childhood vaccination priority decisions, however ascribing relative weights to any of the qualitative preferences is not possible; although this may be a fruitful area of future work. Indeed, the attributes identified during this qualitative study will be used to develop a discrete choice experiment, which will be used to capture UK population preference weights to help inform the future prioritisation of childhood vaccination programmes. This approach will identify the ‘weighting’[41] that members of the wider UK public ascribe to the attributes identified as part of this study. This proposed research will help determine whether to, and how, policy makers should include such preferences in decision making. Such findings will be used to inform discussions around the development and implementation of improved cost-effectiveness approaches for evaluating childhood vaccination programmes.

## Supporting information

S1 File Qualitative interview topic guide. (DOCX)

S2 File Update to the qualitative interview topic guide. (DOCX)

S3 File Additional descriptive characteristics of participants. (DOCX)
