# Supplementary material for: The views of the general public on prioritising vaccination programmes against childhood diseases: A qualitative study
Source: PLoS One. 2018 Jun 13;13(6):e0197374. doi: 10.1371/journal.pone.0197374 (PMC5999095; doi:10.1371/journal.pone.0197374)
Supplement: S1 File — (DOCX) [file pone.0197374.s001.docx]

**Supplementary Information (S1 File) for:**

**The views of the general public on prioritising vaccination programmes against childhood diseases: A qualitative study.**

**Gemma Lasseter^*^, Hareth Al-Janabi, Caroline L Trotter, Fran E Carroll and Hannah Christensen.**

*Corresponding author

E-mail: gemma.lasseter@bristol.ac.uk

**S1 File: Qualitative interview topic guide**

## Opening script

- **Thank** you for your participation.
- **Introductions** - premise of the interview, format and duration.
- **Participation is voluntary**; you can withdraw, take a break or decline to respond at any point.
- **Conversation will be audio recorded** and, on starting the digital recorder, the participant will be asked to verbally confirm that they are still happy to take part in the interview; verbal consent will be recorded.
- **Any questions?**
- **Review information sheet, answer participant’s questions and complete consent form.**

## Background information and general discussion

**Personal/family vaccination history**

1. Can you tell me what experience you/your family had had with vaccinations?
   - **Prompt** with vaccination schedule

**Specific vaccinations of children in the family**

1. Do you have children?
2. If appropriate – can you tell me what sort of vaccinations have they had?

**Things to remember when participants are unwilling to make a rationing decision:**

- Where should we draw the line?
- Fairness – how do you (the participant) balance that, where should we draw the line?
- If it is not possible to offer a vaccination everyone, who should be prioritised?
- Balancing the risk – e.g. 1 individual versus 80,000 people coming down with an infection.

## Vignette example 1:

**I'm going to read a couple of examples of different infections now and then I'm going to ask you for your opinions. Please stop me if you have any questions:**

| **A 16-year-old boy visits his GP with a fever, which he reports having for three days, and swelling on his face that started in the last day. He has not had any recent problems with his teeth. On examination, the doctor notices that the boy has a large tender swelling on the left side of his face and is found to be generally unwell.**  **The GP suggests pain killers, bed rest and drinking plenty of water. The patient was also told to recontact their GP if his symptoms do not improve after seven days, or if they suddenly got worse.**  **Thankfully the fever subsides within seven days and the boy has no lasting side-effects.** |
| --- |

1. Thinking about this example, if a vaccine was available for this infection do you think it should be offered? Why?
   - **Probe** for their general attitudes: age of the patient, severity of the disease and potential long-term complications.

**Severity of infection**

1. If we think about this example, does the severity of the infection affect your decision about whether a vaccination should be made available?
   - **Probe** for different levels of severity, moderate or mild.
   - **What do participants consider to be severe/mild?** Symptoms, time-off, hospitalisation, scale of illness, treatable with antibiotics (milder).

**Ease of transmission**

1. Does how easy it was to catch have any impact on whether you think a vaccination should be made available?
   - **Probe** for the impact of low vs. highly contagious infections.

**Prevalence of vaccine preventable infections**

1. If this type of infection was very rare, would this affect your decision about whether a vaccination should be made available? Why?
2. What if there was no other treatment for this infection (e.g. antibiotics), would that affect your decision?

| **Now, try to imagine that the boy passes the same infection on to his father, however the side-effects are much more serious in adults. His Father becomes very ill and is admitted to hospital, he is treated for serious brain problems for several weeks and eventually makes a good recovery, but due to the infection has long term hearing lose.** |
| --- |

1. Does this affect your opinion about whether a vaccine should be available for this infection? Why do you say that?
   - **Probe** for severity, ease of transmission and prevalence.

## Vignette example 2:

| **A 9-month-old girl is brought into A&E with a fever, vomiting and diarrhoea that she has had for the last 3 days. Mum feels that the child has not been herself for the last few days and seems irritable most of the time. On examination by the doctor the infant has a fever and a rash on her feet and lower legs that does not lighten when pressure is applied.**  **The doctor makes a decision to start the child on antibiotics. In the next 24 hours, the infant condition worsens despite excellent care and she is transferred to the intensive care unit, where she is put on life support. The rash spreads and several toes turn purple, a sign that they may need to be amputated in the future.** |
| --- |

1. Thinking about this example, if a vaccine was available for this infection do you think it should be offered? Why?
   - **Probe** for their general attitudes: age of the patient, severity of the disease and potential long-term complications.

**Severity of infection**

1. If we think about this example, does the severity of the infection affect your decision about whether a vaccine should be made available?
   - **Probe** for different levels of severity, moderate or mild.
   - **What do participants consider to be severe/mild?** Symptoms, time-off, hospitalisation, scale of illness, treatable with antibiotics (milder).

**Ease of transmission**

1. Does how easy it was to catch have any impact on whether you think a vaccine should be made available?
   - **Probe** for the impact of low vs. highly contagious infections.

**Prevalence of vaccine preventable infections**

1. If this type of infection was very rare, would this affect your decision about whether a vaccine should be made available? Why?
2. What about if this type of infection was on the decline, do you think that should affect whether a vaccine was made available.
3. What if there was no other treatment for this infection (e.g. antibiotics), would that affect your decision?

### Herd immunity example (via flashcards):

I have another example, which I'm going to explain using these flash cards....


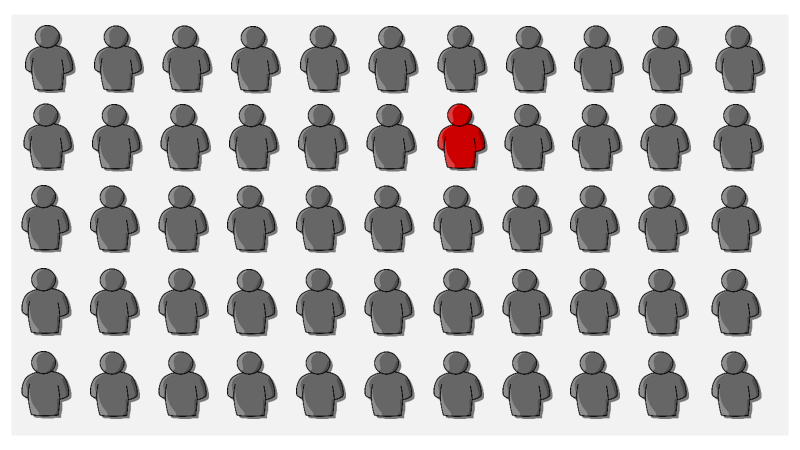
**
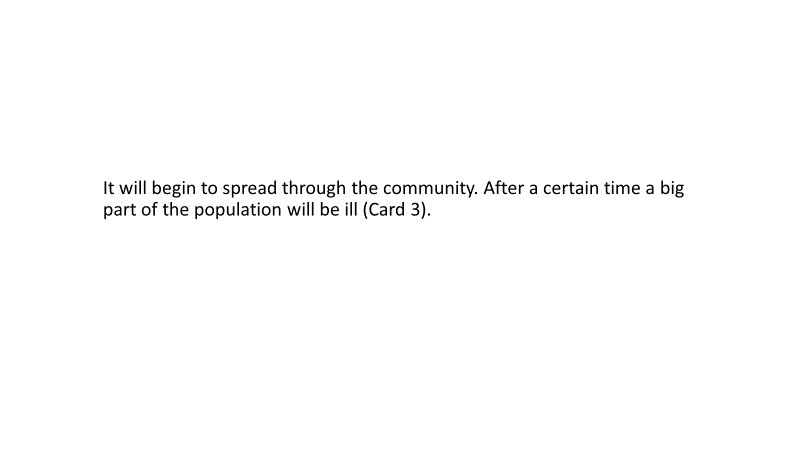
**
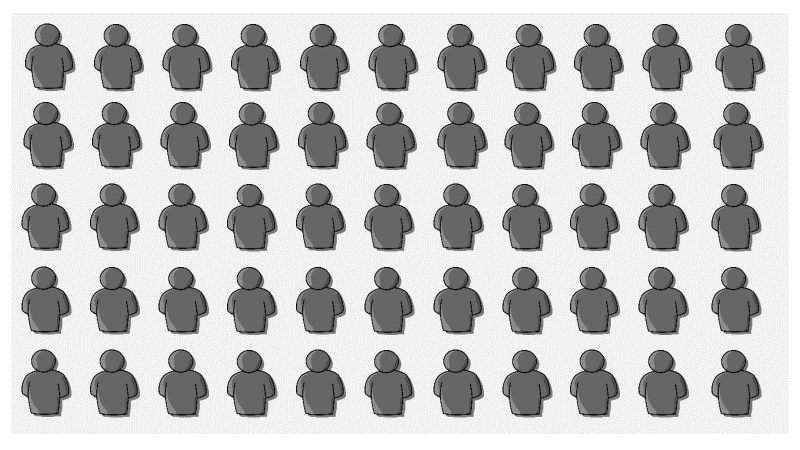

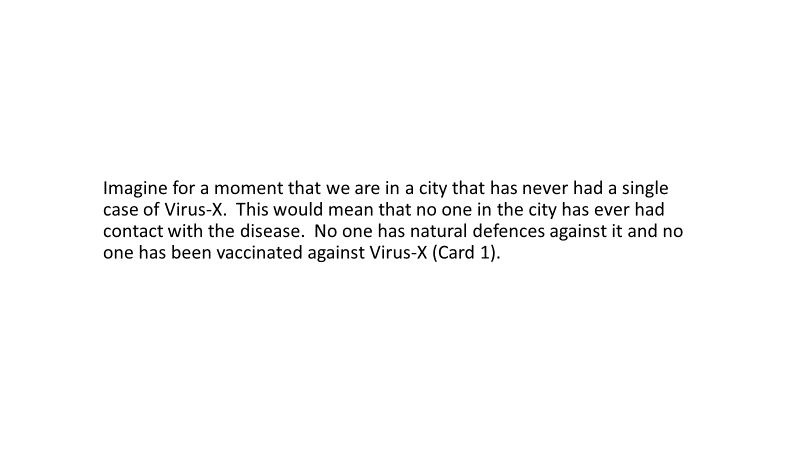

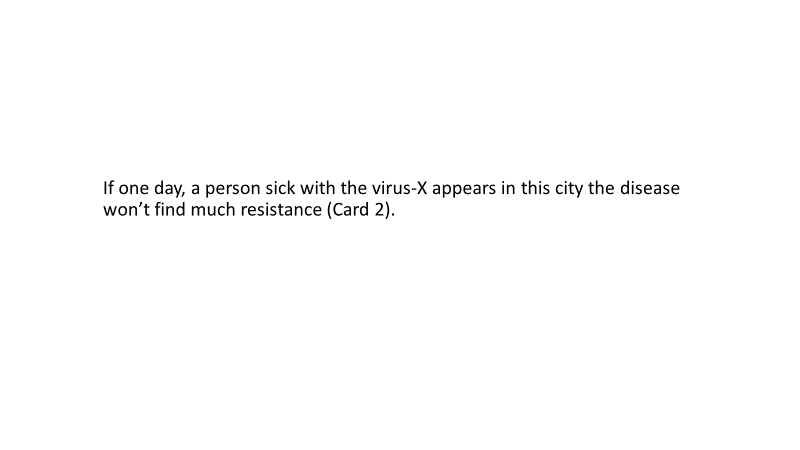
**
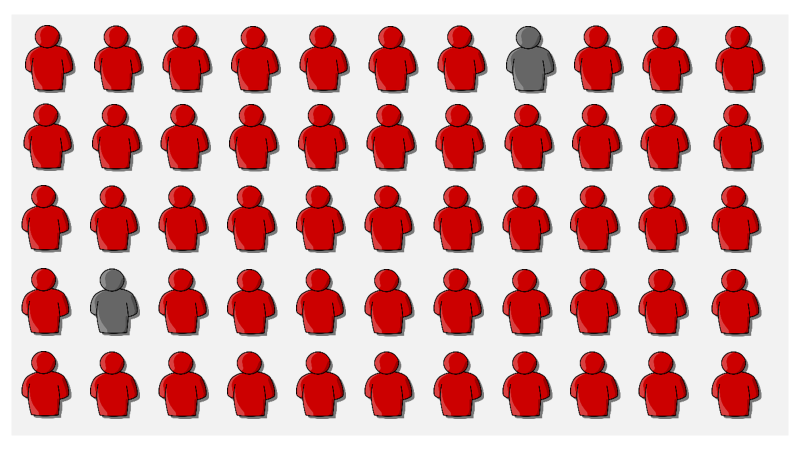

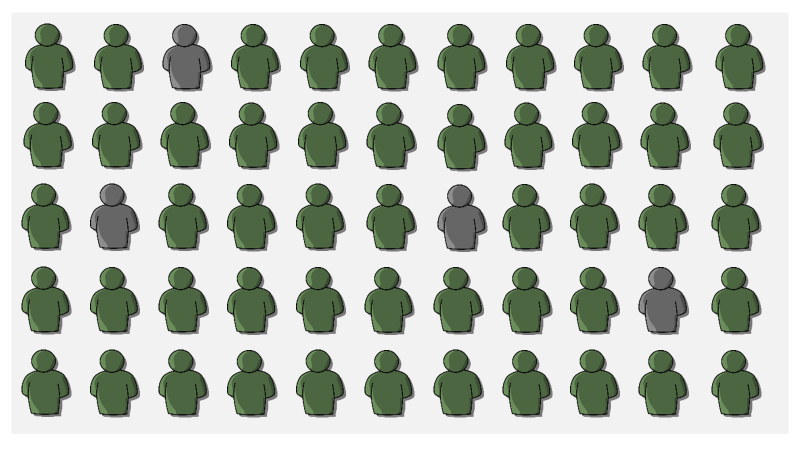
**

**
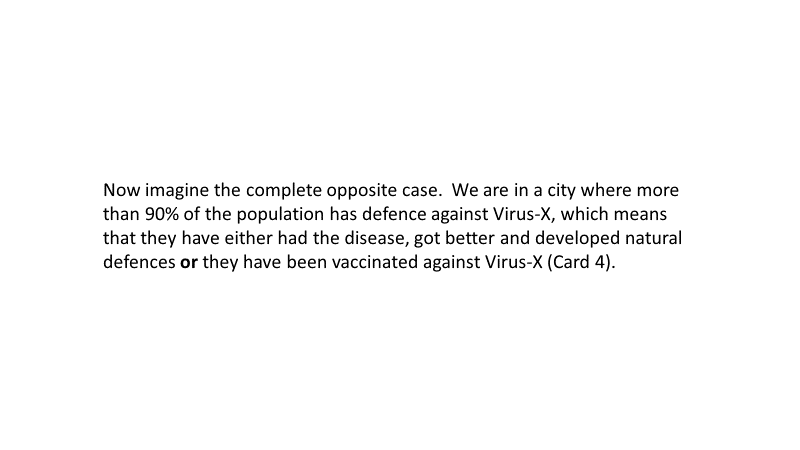
**

**
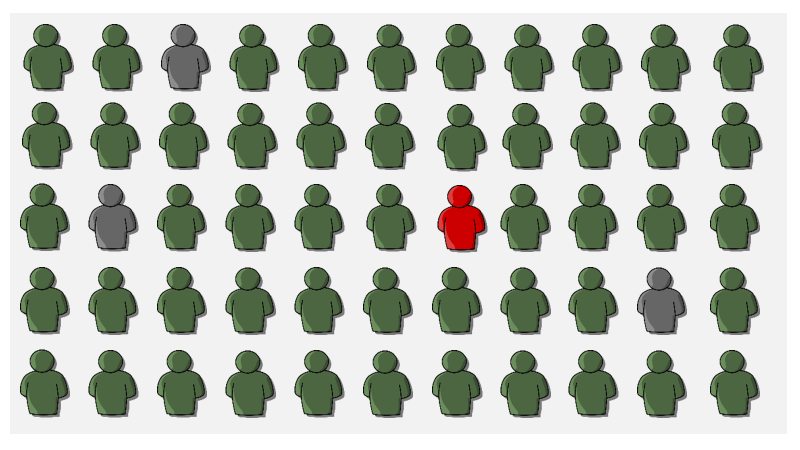

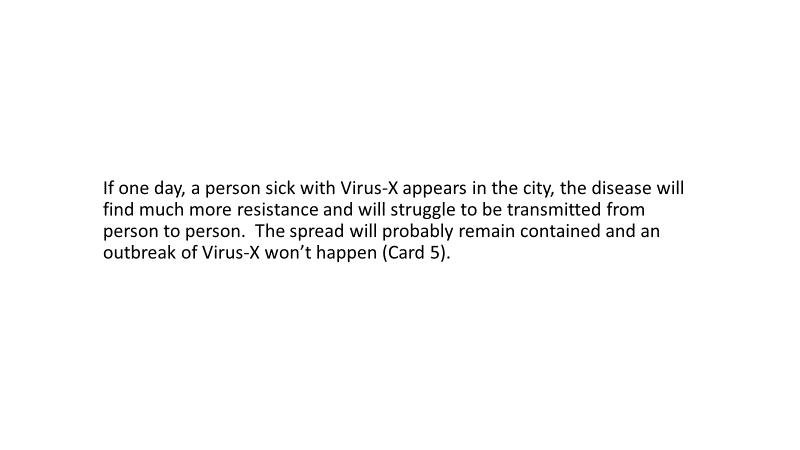
**

**
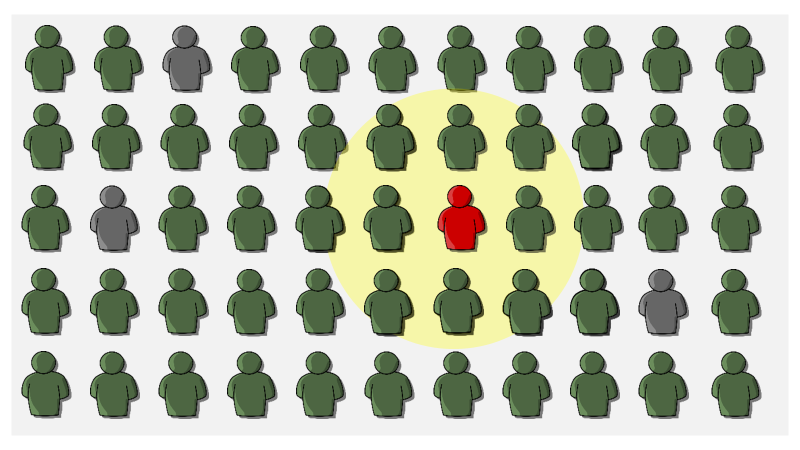

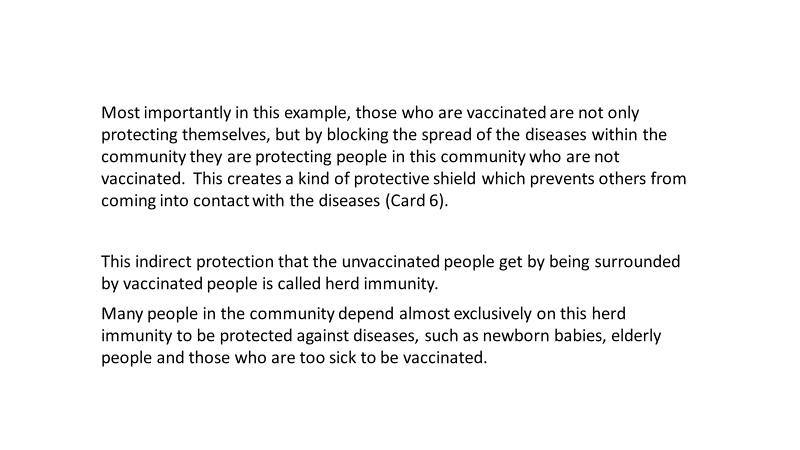
**

| **Herd immunity questions**  Thinking about the information introduced using these flash cards…   1. Is herd immunity something that you have come across before? 2. What do you think about it (e.g. having a vaccination to protect others and not necessarily yourself)?    - **Example** - smallpox eradication. 3. Some vaccinations do not result in herd immunity, as the infection cannot be spread from person to person (such as tetanus) - would this affect your decision to get vaccinated? Why?    - **Probe -** preference for vaccination that confers protection to the individual, vs. willingness to have a vaccination because it protects others. |
| --- |

## Characteristics of health gains example (via flashcards):

Next, I would like to introduce an example about the idea of health gains, which will be explained using the following flash cards:


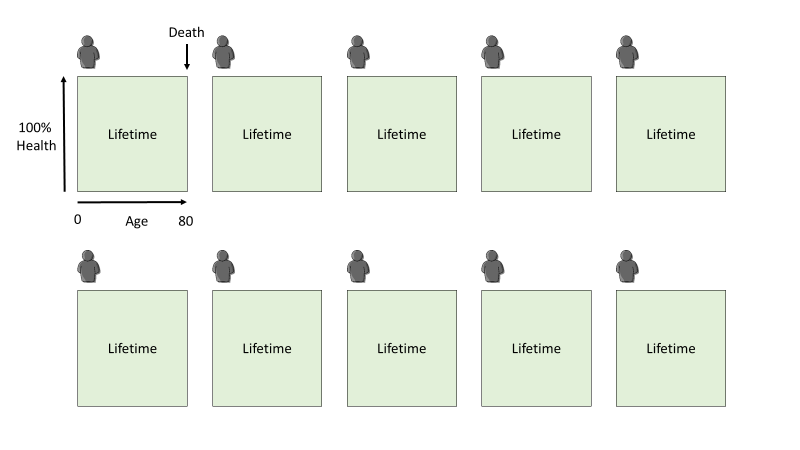

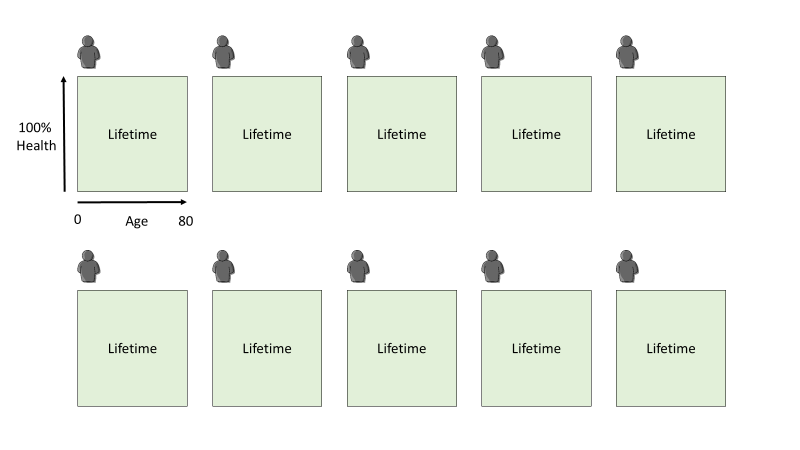

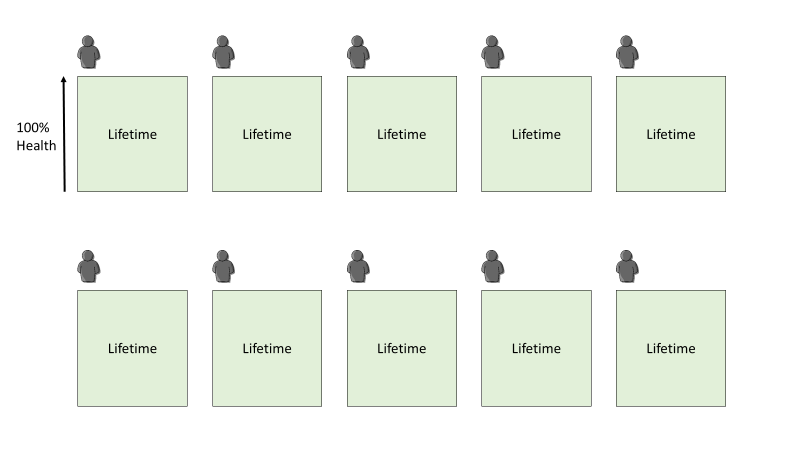

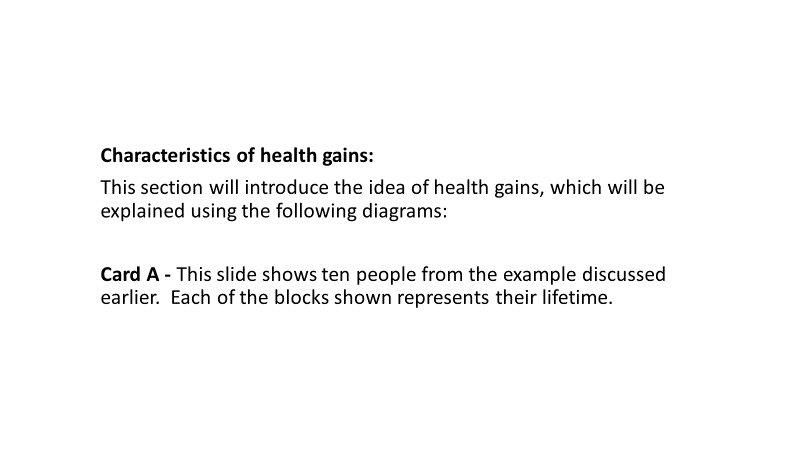

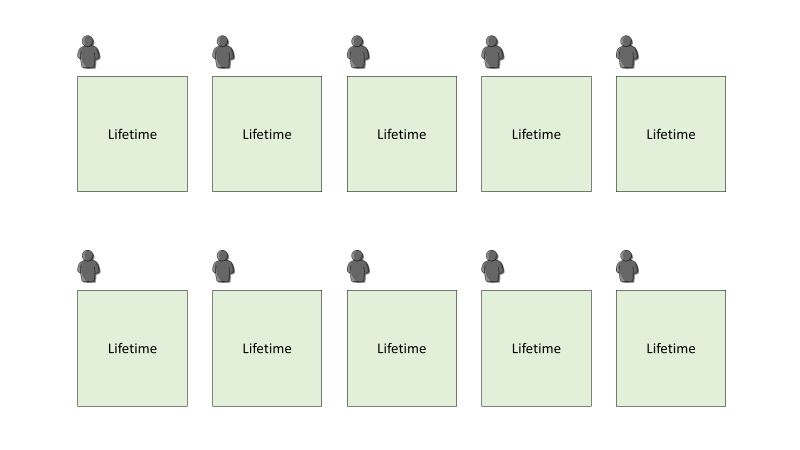

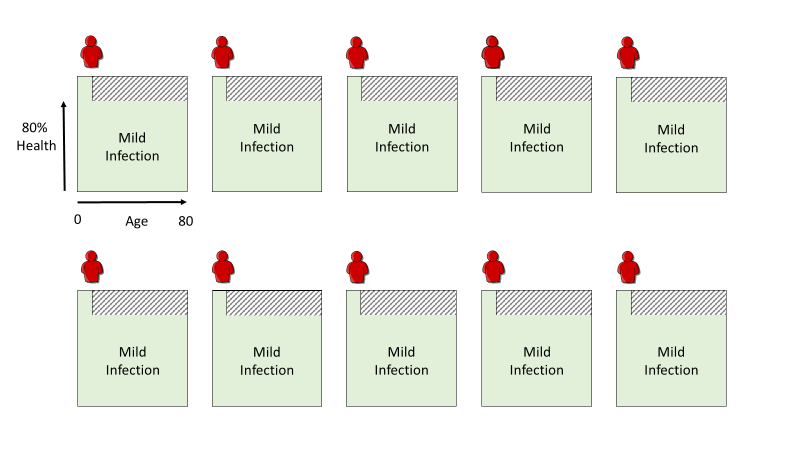


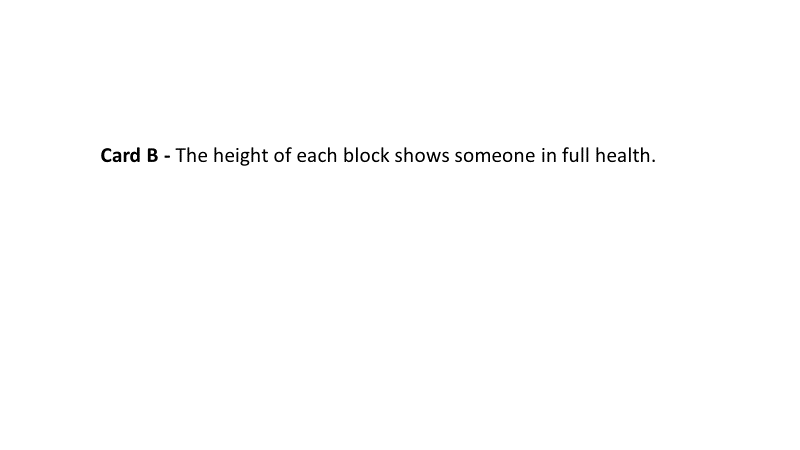


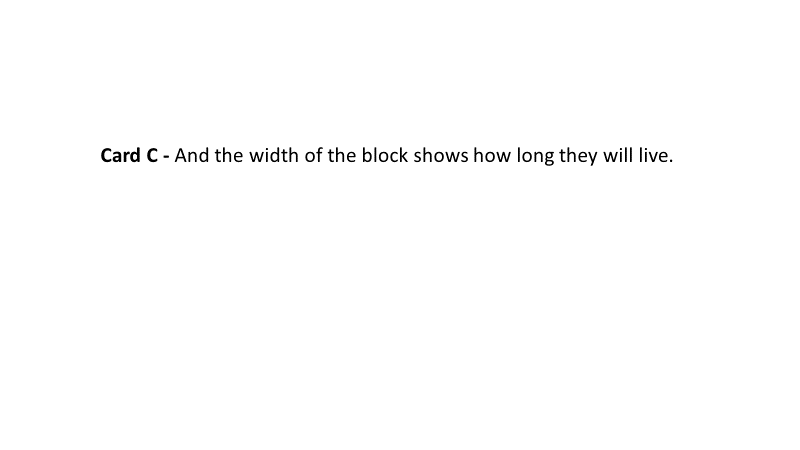


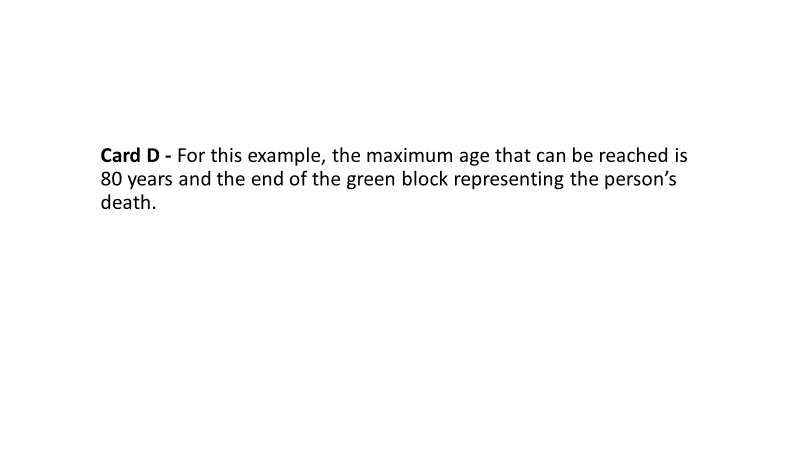


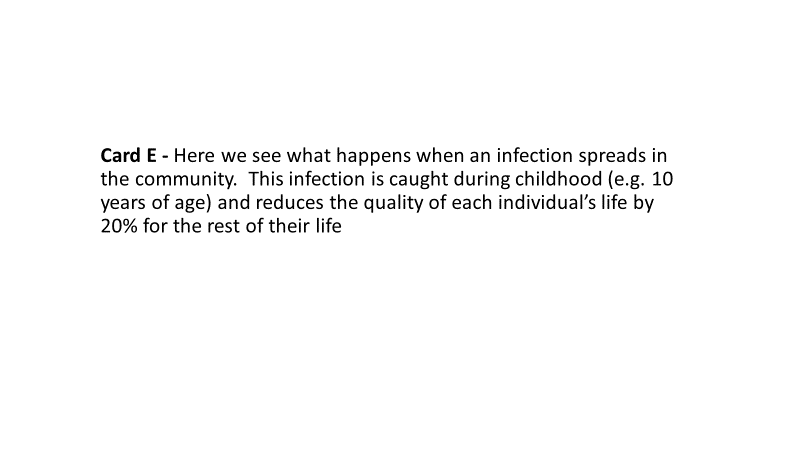


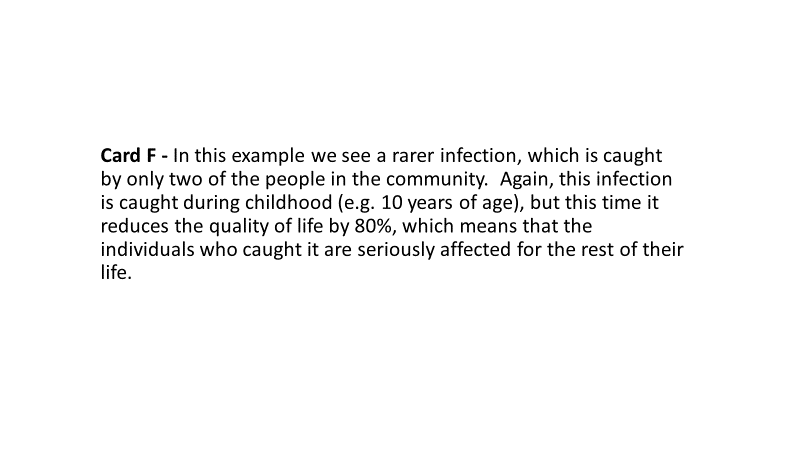

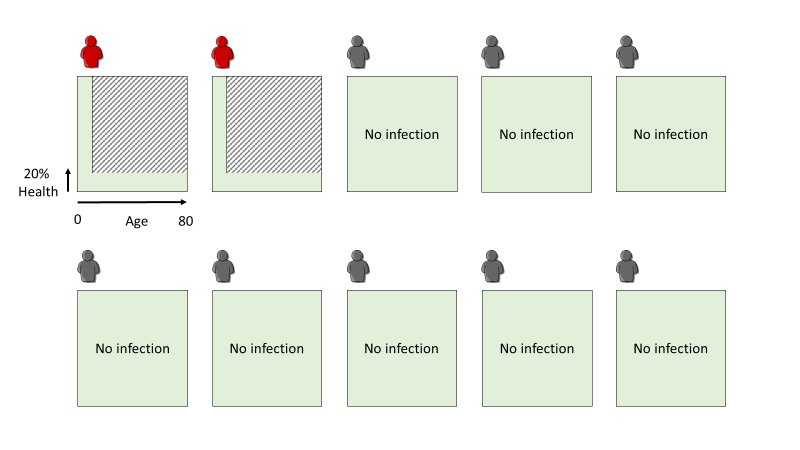

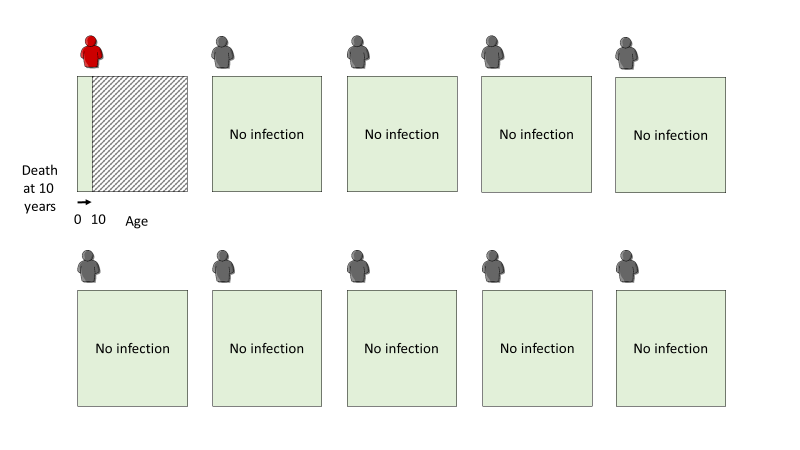

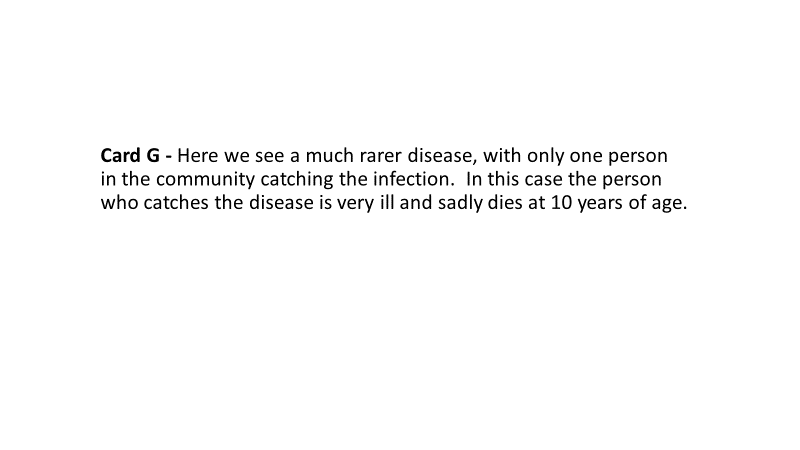


| **Characteristics of the health gain questions:**  These are just examples, but for our discussion they are a useful way to help us think about health and how infections might affect our health during our lifetimes.  With these examples in mind, I want you to think about vaccinations that could reduce the chances of getting an infection that might reduce an individual's overall health (reshow Figures E & F), or reduce the chances of getting an infection that would cause an early death (reshow Figure G).   1. So, what do you think is the most important:    - Providing a vaccination that would have a small health benefit for many people in the community (Figure E)?    - Providing a vaccination that would have a large health benefit, but only for a few in the community (Figure F)?    - Providing a vaccination that would be potentially lifesaving, but only for a very small number of people in the community (Figure G)? 2. **Threshold level** -- what level of health gain do participants consider as important?    - **Probe** - rather **quality of life** -- small (e.g. 10%) vs. large (e.g. 50%)   - or **increase in life expectancy** -- little (e.g. 2 years) vs. large (e.g. 20y)   1. Thinking about **herd immunity** again, **what if the health gain was shared** between many people in the community, would this affect how you felt about a vaccination?    - **Probe** - small health gain for many individuals vs. large health gain for fewer individuals. 2. What if the vaccination actually resulted in **side-effects** that reduced someone's health or life expectancy?    - **Probe -** minor health reduction (e.g. painful arm, malaise, fever, allergic reaction) to more serious health issues (e.g. learning difficulties) | |
| --- | --- |
| Characteristics of health rationing: Now were going to move on and talk about health rationing.  **“The cost of providing health services is growing because of factors like the ageing population, cost of new drugs and lifestyle issues like obesity. This means that some health interventions, like vaccinations, cannot be offered to everyone and must be rationed.”**   1. If we think about rationing vaccinations, do you think there are certain socio-economic groups that should be given priority?   Probe with following example:  **“What if we had two vaccines available, but only had the resources to fund one. Imagine the two vaccines are equally effective (in terms of lives saved or avoiding disability) and the same price, but one prevents an illness that is more common in lower socio-economic (poorer) groups and one prevents an illness that is more common in higher socio-economic (wealthier) groups. Which should we fund and why?"**  If the participant chooses:   1. **Lower SE group** -- because more disadvantaged & should be cared for by the ‘state’? 2. **Higher SE group** -- because they're more economically productive and therefore the economic benefit of this vaccine might be greater? 3. **Can’t choose** – A reasonable response.    1. **Other Probe** – Don’t just ask these, weave them into the conversation ---**Work status/ability to work, household** **income** or **education**. 4. If we think back to the example about rationing vaccinations, do you think there are other people that should be given priority?    1. **Probe - Presence of dependents**: none, children, other individual that requires informal care and so on.    2. **Probe -** **Caring burden or spread of diseases**: Which is the most important?   **Other topics to keep in mind:**   1. **Prevention is better than cure** – **but why?** Cheaper? Avoid illness? Impact on family? 2. **Experts decide about vaccines** – is this OK, would you rather have a debate? 3. **Charging for vaccines** -- making the vaccine available but at a cost? 4. **Lifestyle/culpability for illness** -- self-induced illness and so on. 5. **Fair innings** -- prioritising those individuals that haven't yet had a fair innings in their life over those that have (e.g. young vs. elderly)    1. **Probe** – **Age:** So would you value vaccinations differently if they were given to young children (≤ 1 year), older children (1-10 years), elderly adults (≥ 75 years) – why?    2. **Remaining life expectancy** -- with and without consideration of age. 6. **Peace of mind** - Thinking beyond the health benefits provided by a vaccine, do you think that the ‘peace of mind’ you get from having a vaccination is important? |  |
