# Supplementary material for: The views of the general public on prioritising vaccination programmes against childhood diseases: A qualitative study
Source: PLoS One. 2018 Jun 13;13(6):e0197374. doi: 10.1371/journal.pone.0197374 (PMC5999095; doi:10.1371/journal.pone.0197374)
Supplement: S2 File — (DOCX) [file pone.0197374.s002.docx]

**Supplementary Information (S2 File) for**

**The views of the general public on prioritising vaccination programmes against childhood diseases: A qualitative study.**

**Gemma Lasseter^*^, Hareth Al-Janabi, Caroline L Trotter, Fran E Carroll and Hannah Christensen.**

*Corresponding author

E-mail: gemma.lasseter@bristol.ac.uk

**S2 File: Update to the qualitative interview topic guide**

The examples outline below have been developing using the results from some of the early qualitative interviews.

- Some of these examples were preferred by the majority of participants and these have been highlighted throughout the document (for the interviewer only).
- Other examples have been included for discussion only, as no clear conclusions have been reached.
- Only the examples in the boxes should be provided to the interview participants in order to gauge their overall opinions of the proposed attributes and levels.

|  |
| --- |

**Attribute:** Age group (preferred)

Age range (preferred)

Target age group

Age category

Life stage

Groups of people

**Attribute levels:** See examples 1 and 2 below.

**Example 1:** Three childhood and one adult example

| **Age group** |
| --- |
| 1 year old or less |
| 2 to 11 years |
| 12 to 17 years |
| 65 years and older |

**Example 2:** Two childhood and two adult examples

| **Age group** |
| --- |
| Less than 2 years of age |
| 2 to 17 years |
| 18 to 64 years |
| 65 years and older |

- Some participants felt that the ‘1 year old or less’ age group was important to keep as a separate category, as children in this age group were more ‘vulnerable’. Whilst others felt that ‘Less than 2 years of age’ was sufficient, as this was when most children received the majority of their vaccinations.
- We wondered whether participants might be concerned about the quantity of individuals that would be included in each age group and whether this might influence their decision in the final questionnaire. However, when specifically asked about this none of the participants considered this to be an issue.

**Attribute name:** Severity (preferred)

Symptoms

How severe?

What happens if not vaccinated?

**Explanation at start of questionnaire:**

Refers to the symptoms of a vaccine preventable disease as experienced by an ill child. It is classified into four levels: mild, moderate, severe and very severe.

**Attribute levels:**

- Mild, moderate, severe and very severe (Preferred – see below)
- Other potential levels that were mentioned for classifying severe/very severe:
  - Possibly life-limiting
  - Possible long-lasting effects
  - Possibly life threatening
  - Death

**Example 1**:

- **Mild:** general discomfort, tiredness or cold-like symptoms. Child may make a full recovery.
- **Moderate:** fever, headache, muscle and joint pains, frequent diarrhoea or vomiting. Child may make a full recovery.
- **Severe:** high fever, severe headaches, temporary unconsciousness, tremors or paralysis. Child may be left with long-term physical or emotional (mental) disability.
- **Very severe:** high fever, limb loss, brain damage or organ failure. Child will most likely have long-term physical or emotional (mental) disability and may die as a result of the disease.

**Example 2**:

- **Mild:** Every year there are approximately 130,000 childhood cases in the UK, with 13,000 children admitted to hospital and four deaths.
- **Moderate:** Every year there are approximately 520,000 childhood cases in the UK, with 52,000 children admitted to hospital and 20 deaths.
- **Severe**: Every year there are approximately 3,000 childhood cases in the UK, with 500 children admitted to hospital, 30 children developed serious long-lasting complications and 2 deaths.
- **Very severe:** Every year there are approximately 750 childhood cases in the UK, with 750 children admitted to hospital, 300 children developed serious long-lasting complications and 50 deaths.

**Attribute name:** Prevalence (preferred)

How common?

Incidence

How will it impact on you?

**Explanation at start of questionnaire:**

Refers to the regularity of vaccine preventable disease in UK children. It is classified into four levels: very common, common, uncommon and rare.

**Attribute levels:** Very common, common, uncommon and rare.

**Example 1:**

- **Very common** affects 1 in 10 children
- **Common** affects 1 in 100 children
- **Uncommon** affects 1 in 1,000 children
- **Rare** affects 1 in 10,000 children

**Example 2:**

- **Very common** affects 1000 in 10,000 children
- **Common** affects 100 in 10,000 children
- **Uncommon** affects 10 in 10,000 children
- **Rare** affects 1 in 10,000 children

**Example 3:**

(Please note: The example below was for discussion only, as participants were asked to colour in their own bar charts to illustrate how they might estimate ‘very common’ to ‘rare’. Irrespective of this, all participants preferred Example 1.

**Very common**

**Common**

**Uncommon**

**Rare**

**Attribute name:** Care impact

Care burden

Care responsibility

**Explanation at start of questionnaire:**

When a child gets a disease, this can have an impact on the quality of life (health and wellbeing) of their close family and friends because these people are concerned and care for the child during and after their illness.

**Attribute levels:** Quality of life (health and wellbeing) 5%, 20%, 35% and 50%

**Example 1:**

**Vaccination programme A would prevent the following disease:**

| Child with illness, quality of life reduced by 35% | Family and friends who care for the ill child, quality of life reduced by 5% |
| --- | --- |

**Vaccination Programme B would prevent the following disease:**

| Child with illness, quality of life reduced by 20% | Family and friends who care for the ill child, quality of life reduced by 50% |
| --- | --- |

Would you choose **Vaccination programme A or B?**

- In general participants felt uncomfortable with using percentages to describe care impact, as this did not seem to adequately convey what would actually happen in the family setting.
- With this in mind, some individuals talked about having qualitative examples of care impact, like those used for ‘Severity’:
  - **Mild impact**: The disease should not impact on your day to day life, but you may need to make small adjustments to care for the ill child.
  - **Moderate impact**: You may need to make short to medium term adjustments to get your child back on the road to recovery.
  - **Severe impact**: May need to take longer term adjustments to lifestyle to get your child back up to a percentage of recovery, although the child will never return to 100% and so this may have an impact on your long-term family life.
  - **Very severe**: Definite change to lifestyle would be required and this would impact on your long-term family life.

**Attribute name:** Socio-economic status

Social advantage

Social disadvantage

Social health benefit

Social class

**Explanation at start of questionnaire:**

Refers to the percentage health benefit that each population group (socially advantaged versus socially disadvantaged) gets from a vaccination.

**Attribute levels:** To be determined

**Example 1:** Imagine that you are asked to choose between two different vaccination programmes. Both prevent disease and so offer health benefits. There is 100% health benefit available from each vaccination programme:

**Vaccination A:** For Vaccination Programme A, 50% of these health benefit goes to socially advantaged individuals and 50% to disadvantaged individuals in society.


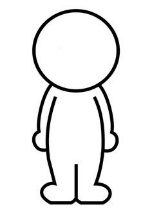

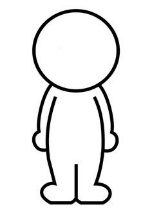


50%

50%

Socially

disadvantaged

Socially

advantaged

**Vaccination B:**  For Vaccination Programme B, 88% of the health benefit goes to socially advantaged individuals and 18% to disadvantaged individuals in society.


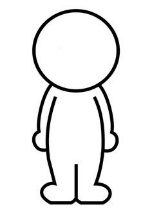

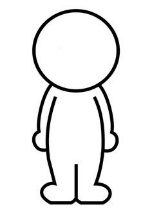


12%

88%

Socially

advantaged

Socially

disadvantaged

Which would you choose, **Vaccination programme A or B?**

- For this last example previous participants talked about socio-economic status in terms of economic/financial status rather than health status. When asked to consider the difference in health status between social groups, participants considered these two things to be interlinked and undistinguishable.
- Is there confusion about the number of people in each group?
- Do the percentages make sense to participants?
