# Supplementary material for: The views of the general public on prioritising vaccination programmes against childhood diseases: A qualitative study
Source: PLoS One. 2018 Jun 13;13(6):e0197374. doi: 10.1371/journal.pone.0197374 (PMC5999095; doi:10.1371/journal.pone.0197374)
Supplement: S3 File — (DOCX) [file pone.0197374.s003.docx]

**Supplementary Information (S3 File) for:**

**The views of the general public on prioritising vaccination programmes against childhood diseases: A qualitative study.**

**Gemma Lasseter^*^, Hareth Al-Janabi, Caroline L Trotter, Fran E Carroll and Hannah Christensen.**

*Corresponding author

E-mail: gemma.lasseter@bristol.ac.uk

S3 File: Additional descriptive characteristics of participants (n=21)

| **Characteristics** | **Number** |
| --- | --- |
| Employment status |  |
| In active paid work | 13 |
| Retired | 7 |
| Other | 1 |
| Religion |  |
| Christian | 9 |
| None | 12 |
| Health in general |  |
| Fair | 5 |
| Good | 9 |
| Very good | 7 |
| Previous academic research participation |  |
| Yes | 8 |
| No | 13 |
